# Potential risk of occupational exposure to respirable crystalline silica in Saudi Arabia: a comprehensive review of the current status and future challenges

**DOI:** 10.3389/fpubh.2026.1918362

**Published:** 2026-10-05

**Authors:** Salman Alquwayi

**Affiliations:** Department of Environmental Health, College of Public Health, Imam Abdulrahman Bin Faisal University, Dammam, Saudi Arabia

**Keywords:** occupational exposure, public policy development, respirable crystalline silica (RCS), silica dust, silica regulation, silicosis, Saudi Arabia

## Abstract

Respirable crystalline silica (RCS) is a major occupational health hazard associated with silicosis, chronic obstructive pulmonary disease (COPD), lung cancer, autoimmune diseases, and other systemic disorders. Saudi Arabia is undergoing rapid industrial expansion in sectors with substantial potential for RCS exposure, including mining, construction, cement production, glass manufacturing, oil and gas, engineered stone fabrication, and photovoltaic manufacturing. This review evaluates the potential risk of occupational RCS exposure in Saudi Arabia by integrating evidence from the scientific literature, government reports, labor and mining statistics, mineralogical data, and regulatory documents. The novel silica Hazard triangle framework was applied to assess three principal determinants of occupational exposure: workforce distribution, silica-containing materials, and high-risk industrial activities. Saudi Arabia possesses extensive reserves of high-purity silica sand, and continued expansion of mining and downstream silica-based industries may increase occupational exposure potential. The kingdom’s arid climate may also contribute to dust generation and increase the occupational exposure of RCS. Although documented cases of silicosis confirm that silica-related disease exists in Saudi Arabia, published evidence on occupational exposure assessment, epidemiology, and health surveillance remains limited. International regulatory developments demonstrate the importance of health-based occupational exposure limits (OELs), exposure monitoring, engineering controls, and medical surveillance. Current Saudi labor law, National Council for occupational safety and health (NCOSH) policies, and General Organization for Social Insurance (GOSI) occupational disease frameworks provide an important legal and institutional foundation for the future development of silica-specific occupational health regulations. This review identifies important knowledge gaps and highlights the need for comprehensive exposure assessment, strengthened industrial hygiene capacity, and evidence-based regulatory strategies to reduce the future burden of silica-related diseases while supporting sustainable industrial development in Saudi Arabia.

## Graphical Abstract

**Figure:**
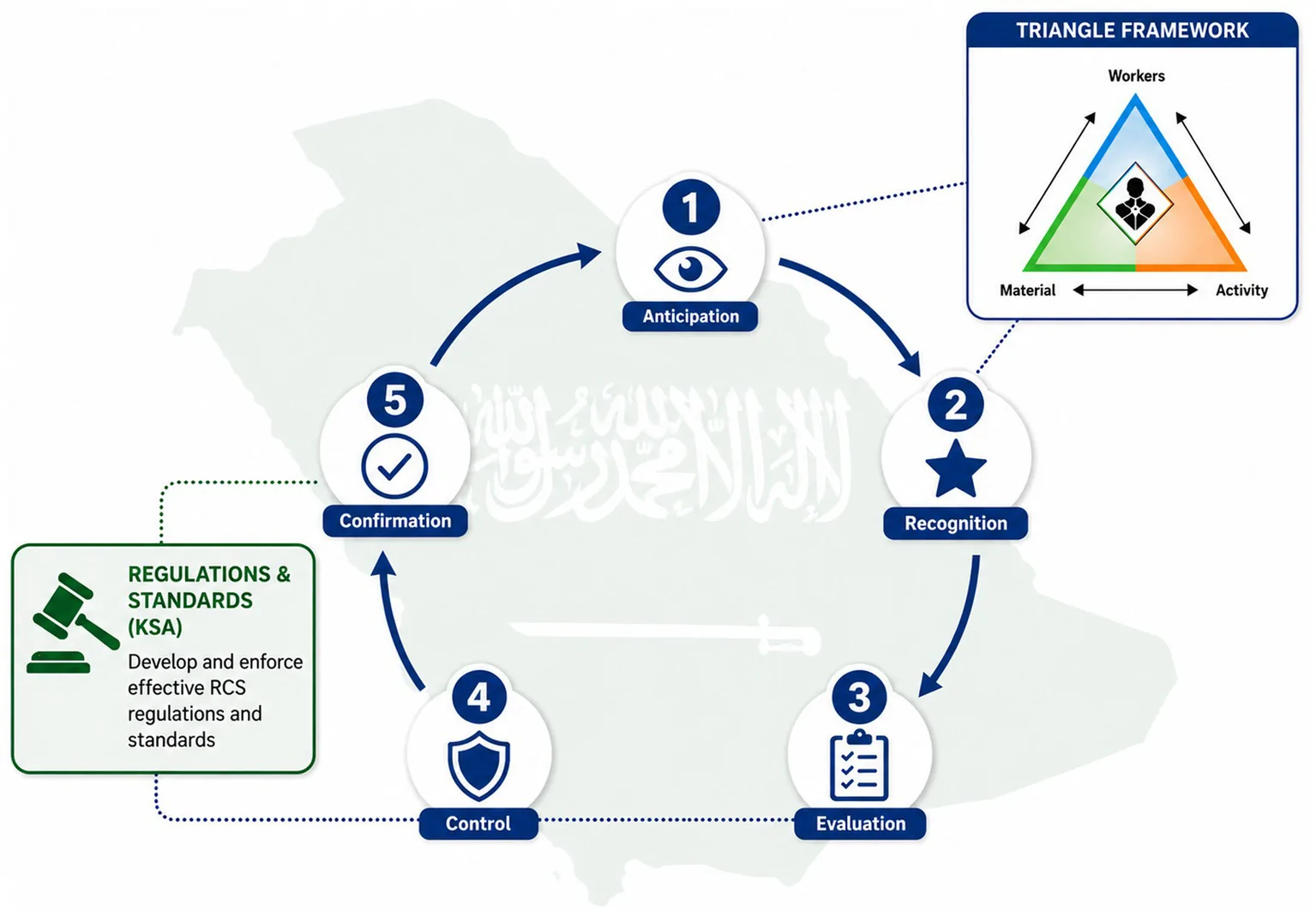

## 1 Introduction

Globally, respirable crystalline silica (RCS) is a serious occupational and environmental health issue. A recent public health study investigated the burden of RCS in 204 countries and territories between 1990 and 2021. Although global incidence, mortality, and disability-adjusted life years (DALYs) associated with silicosis have shown an overall upward trend, the corresponding age-standardized rates (ASRs) declined over the same period. These trends highlight that although the global number of people affected by silicosis has increased, the age-standardized mortality rate from silicosis has declined. Some countries, such as South Africa, China, and Chile, experience a higher burden of silicosis due to high recorded age-standardized incidence and DALY rates exceeding the global average (1). Importantly, the lack of silicosis case reports or surveillance data in some countries does not necessarily indicate the absence of RCS exposure or disease burden. The actual burden of silicosis may be underestimated due to insufficient occupational health surveillance systems, underreporting, inadequate diagnostic capacity, and the lack of national registries. Occupational exposure may therefore remain undocumented despite ongoing silica-generating industrial activities in some countries.

The Global Burden of Disease (GBD) of silicosis accounted for more than 2,000 incident cases, over 12,900 deaths, and approximately 650,000 DALYs worldwide in 2019 (1). This major global public health issue emphasizes the urgent need for action. Approximately 230 million people are exposed to RCS annually, with 40.5 million working in the mining industry. In the United States and Europe, around 2 million people are exposed to RCS each year. Cases of silicosis are particularly observed in middle- and upper-middle-income regions (1). In the Bay of Cádiz in southern Spain, 106 workers were diagnosed with artificial stone silicosis between 2009 and 2018. Despite removal from further silica exposure, 56% showed radiographic disease progression, highlighting the aggressive nature of artificial stone-associated silicosis (2).

Amorphous silica is generally considered less hazardous than crystalline silica when inhaled. This could be due to the surface reactivity of crystalline silica, which promotes long-term lung inflammation and tissue damage. The predominant crystalline silica associated with environmental and occupational exposure is quartz. On the other hand, tridymite and cristobalite are comparatively less common and can be found in high temperature industrial processes (3). The surface reactivity of crystalline silica promotes alveolar inflammation, macrophage activation, fibrotic signaling, and epithelial injury. The most well established health consequences of occupational RCS exposure include silicosis, lung cancer, chronic obstructive pulmonary disease (COPD), and increased susceptibility to tuberculosis (4). Similarly, epidemiological evidence also supports associations between RCS exposure and certain autoimmune diseases (5). In addition to the crystalline structure, particle size also can be a determinant of the toxicity. RCS consists of crystalline silica particles within the respirable aerosol fraction that are sufficiently small to penetrate the gas-exchange region of the lungs. The respirable convention is defined by a sampling efficiency curve with a 50% cut-point (D₅₀) of approximately 4 μm aerodynamic diameter rather than by a fixed upper particle size limit. These small respirable particles can bypass the upper respiratory tract mucociliary escalator and deposit in the alveoli, where long-term retention occurs due to limited phagocytic or mechanical clearance. Deposition in the alveoli enables prolonged particle retention, repeated macrophage injury, and chronic inflammatory and fibrotic responses. The effects of exposure extend beyond COPD and silicosis to include cardiac (6) and renal effects (7); however, the evidence for these outcomes is emerging and remains comparatively more limited, with the evidence base continuing to develop. A case report from China described a coal miner with long-term silica exposure who developed silicosis along with biopsy confirmed IgA nephropathy, highlighting the potential renal implications of silica inhalation (8). Additionally, epidemiological studies indicate that occupational exposure to RCS is associated with an increased risk of systemic autoimmune diseases, including systemic lupus erythematosus, rheumatoid arthritis, and systemic sclerosis (9).

Regional evidence demonstrates that occupational exposure to RCS remains a significant hazard. This is further supported by epidemiological studies. In Egypt, a study of 22 factories across mineral dust related industries, such as stone cutting, ceramics, and glass manufacturing, reported exposure levels exceeding national and international limits, indicating elevated risks of lung cancer and respiratory disease and confirming the widespread presence of RCS in industrial environments (10). Similarly, studies conducted in Iran reported consistent RCS exposures above permissible limits across multiple high-risk sectors, including ceramics, stone cutting, and mining, indicating that workers could develop adverse health effects as a result of the elevated exposure (11–13). These findings confirm that exposure to RCS constitutes a major occupational and public health concern in the region, underscoring the need for stricter exposure control and worker protection strategies. Although these studies provide consistent evidence that silica-generating industries are associated with hazardous occupational exposures, differences in industrial practices and regulatory environments mean that these findings should be interpreted as contextual evidence rather than direct estimates for Saudi Arabia.

Recently, Saudi Arabia has experienced rapid industrial expansion, particularly in construction, oil and gas, cement production, glass manufacturing, photovoltaic (PV) panel production, and a rapidly growing mining sector. Consequently, potential occupational exposure to silica dust has emerged as an important and growing concern requiring urgent evaluation and regulatory attention. Economic data further support the growing industrial relevance of these high-exposure sectors, with mining and quarrying representing the largest contributor to GDP (≈268.4 billion SAR) in 2025-Q3, followed by manufacturing (≈193.74 billion SAR) and construction (≈93.77 billion SAR). These findings highlight the critical economic importance of these dominant sectors, underscoring the increasing importance of evaluating potential RCS exposure within these rapidly expanding industries (14).

Despite the growing importance of these silica-generating industries, published evidence describing occupational RCS exposure and silica-related diseases in Saudi Arabia remains limited. The available literature consists primarily of isolated clinical case reports and descriptive studies rather than comprehensive epidemiological or exposure assessment investigations among workers or surrounding communities. Notably, a previous clinical study titled “Silicosis of the Lung in Saudi Arabia: Role of Traditional Occupations and Environmental Factors” documented and confirmed that silica-related lung disease exists within the Saudi context despite the absence of formal exposure monitoring. The study also highlighted traditional occupational activities and environmental dust as contributing factors to the increase in silicosis cases (15). Investigators confirmed the risk of occupational exposure by examining three cases of advanced silicosis with progressive massive fibrosis among lifelong well diggers and a grave digger. Additionally, 13 reported cases of “desert lung” (simple siliceous pneumoconiosis), mainly among women performing traditional dust-related domestic tasks, supported both occupational and environmental sources of exposure.

Furthermore, a recent analysis of multiple countries estimated that there were 80 verified cases of silicosis per 100,000 people in Saudi Arabia, indicating a non-negligible burden despite the limited national surveillance data (16). While this analysis identified a significant association between silicosis incidence and tuberculosis mortality and disability at the global level, similar associations between silica exposure, silicosis, and tuberculosis have been reported across multiple epidemiological and cohort studies, supporting the biological plausibility of this relationship while recognizing that causality cannot be inferred from observational study design (17, 18). These findings underscore that RCS-related disease is present but likely underrecognized in Saudi Arabia, particularly in the context of expanding activities such as drilling, cutting, grinding, sandblasting, and material handling, which substantially increase the potential for silica dust generation and warrant urgent evaluation and preventive action. This concern may be particularly relevant to the construction sector, where migrant workers constitute approximately 89% of the workforce, many originating from South and Southeast Asian countries. Language barriers, differences in occupational safety training, and other challenges experienced by migrant workers may complicate occupational health surveillance and contribute to the underrecognition or underreporting of chronic occupational diseases such as silicosis (19). Although these reports confirm the occurrence of silica-related disease in Saudi Arabia, they are insufficient to estimate the national burden because comprehensive health surveillance, occupational exposure monitoring, and epidemiological studies remain lacking.

This review highlights the importance of occupational exposure assessment of RCS using the Silica Hazard Triangle framework (see Figure 1) in Saudi Arabia and also highlights the urgent need for a robust regulatory framework to protect workers in high-risk industries. The objective of this review is to characterize the potential for occupational exposure to RCS in Saudi Arabia and identify major evidence gaps by integrating evidence from the scientific literature, government reports, labor statistics, mineralogical data, and regulatory documents. The review applies the Silica Hazard Triangle, a simplified conceptual framework for identifying potential occupational RCS exposure through the convergence of three components: workers, silica-containing materials, and activities capable of generating RCS. The framework is intended for preliminary hazard identification rather than quantitative exposure or health risk assessment. By examining these determinants alongside the available evidence on health outcomes and existing regulatory frameworks, this review aims to identify current knowledge gaps and provide recommendations to support future occupational exposure assessment, research, and policy development in Saudi Arabia. Accordingly, this review is organized into four main components. First, the review methodology is described. Second, the three determinants of occupational exposure are examined using the Silica Hazard Triangle framework in Saudi Arabia. Third, the current occupational exposure standards are reviewed to provide context for the potential occupational burden. Finally, the existing Saudi regulatory framework is examined to identify current strengths, remaining gaps, and priorities for future occupational health policy, exposure assessment, and worker protection.

**Figure 1:**
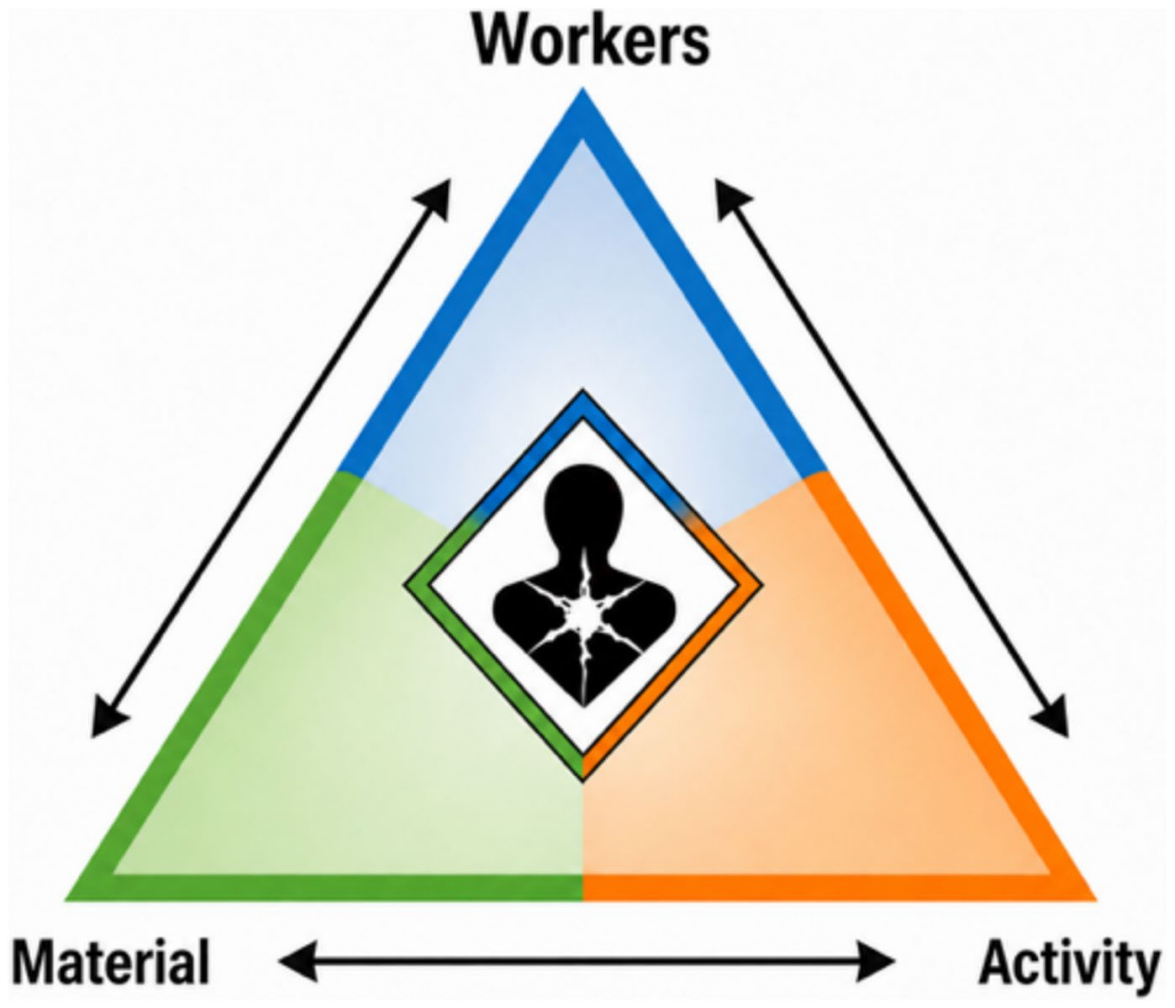
Factors influencing occupational RCS exposure risk: The Silica Hazard Triangle framework.

## 2 Methodology

### 2.1 Review design and search framework

This study employed a narrative review methodology to synthesize the available evidence on potential risk of occupational exposure to RCS in Saudi Arabia. The literature search was structured using the novel Silica Hazard Triangle framework, which considers three interacting determinants of potential occupational RCS exposure: workers, materials, and activities. Rather than treating these components only as a framework for evidence synthesis, they were used to guide the literature search and organization of the retrieved evidence.

The search was conducted using PubMed and Google Scholar through May 23, 2026. The search was conducted primarily in English, with supplementary Arabic language terms used to identify relevant Saudi governmental, legislative, and regulatory documents. In addition to the scientific literature, official Saudi government and institutional sources were searched to obtain information on workforce distribution, mineral resources, occupational health regulations, occupational disease recognition, and national policies. These sources included the General Authority for Statistics (GASTAT), National Council for Occupational Safety and Health (NCOSH), General Organization for Social Insurance (GOSI), Ministry of Human Resources and Social Development (MHRSD), Ministry of Industry and Mineral Resources, Saudi Geological Survey, DataSaudi, and the Small and Medium Enterprises General Authority (Monsha’at).

### 2.2 Search strategy

The literature search was conducted sequentially according to the three components of the Silica Hazard Triangle: workers, materials, and activities. For each component, Saudi specific evidence was searched first. Because Saudi specific occupational RCS literature was limited, the search was subsequently expanded to international peer-reviewed literature to provide scientific context and evidence regarding established exposure pathways.

For the workers component, the search focused on identifying worker populations and occupational sectors in Saudi Arabia with potential for RCS exposure. Search combinations included terms such as (“respirable crystalline silica” OR “RCS” OR “silica exposure”) AND (workers OR occupational) AND (“Saudi Arabia” OR Saudi). The search was subsequently expanded to international peer-reviewed studies using combinations of (“respirable crystalline silica” OR “RCS” OR “silica exposure”) AND (workers OR occupational). Saudi workforce statistics and official employment data were additionally reviewed to characterize the size and distribution of worker populations employed in relevant industrial sectors. These workforce figures were interpreted as indicators of populations with potential risk for occupational exposure and not as estimates of the number of workers with demonstrated RCS exposure.

For the materials component, the search examined the occurrence and characteristics of crystalline silica-containing materials relevant to Saudi Arabia. Search combinations included (“respirable crystalline silica” OR “crystalline silica” OR quartz OR “silica sand”) AND (material OR mineral OR rock OR sand) AND (“Saudi Arabia” OR Saudi). Scientific publications, geological reports, mineral resource assessments, and official governmental Saudi sources were reviewed to characterize silica-containing materials and mineral resources that could contribute to occupational RCS generation when disturbed or processed.

For the activities component, the search focused on industrial processes and occupational tasks capable of generating RCS. Saudi specific search terms included combinations such as (“respirable crystalline silica” OR “RCS” OR “silica dust”) AND (“Saudi Arabia” OR Saudi) AND (“industrial” OR “industry” OR “task” OR “activity” OR “construction” OR “mining” OR “quarrying” OR “drilling” OR “cutting” OR “grinding” OR “crushing” OR “cement” OR “glass” OR “ceramic” OR “engineered stone” OR “oil and gas” OR “hydraulic fracturing” OR “photovoltaic”). Because direct Saudi exposure measurements were limited, international peer-reviewed studies were subsequently searched using similar combinations without the Saudi Arabia restriction. These studies were used to identify established RCS-generating tasks, measured occupational exposure pathways, and industries in which RCS exposure has been documented.

Additional targeted searches were performed when necessary to identify evidence concerning silica-related health outcomes, occupational exposure limits (OELs), exposure monitoring, medical surveillance, and regulatory approaches. Relevant sources from international occupational health and regulatory organizations included the Occupational Safety and Health Administration (OSHA, USA), the Mine Safety and Health Administration (MSHA, USA), the National Institute for Occupational Safety and Health (NIOSH, USA), and the International Labour Organization (ILO).

### 2.3 Eligibility and source selection

Sources were considered eligible when they provided information relevant to at least one component of the Silica Hazard Triangle or to the broader objectives of the review. Eligible sources included peer-reviewed occupational exposure and epidemiological studies, case reports and case series relevant to silica-related disease, review articles, government reports, official workforce and mining statistics, geological and mineralogical publications, occupational health guidelines, and regulatory or legislative documents.

Saudi specific evidence was prioritized when available. International studies were included when Saudi specific evidence was unavailable or insufficient and when they provided relevant information on established RCS exposure pathways, occupational tasks, measured exposure concentrations, silica-related health effects, exposure controls, or regulatory approaches. Findings from international studies were used as contextual evidence and were not interpreted as direct estimates of occupational RCS exposure among workers in Saudi Arabia.

Sources were excluded when they were unrelated to RCS or crystalline silica, did not provide information relevant to the workers, materials, or activities framework or the regulatory and health objectives of the review, or represented duplicate or less authoritative versions of information for which an original peer-reviewed or official source was available.

### 2.4 Evidence synthesis and interpretation

The retrieved evidence was synthesized narratively according to the three components of the Silica Hazard Triangle. The workers component was used to characterize the distribution of worker populations across industries where RCS exposure could potentially occur; the materials component was used to determine whether silica-containing materials are present in relevant Saudi industrial and geological settings; and the activities component was used to identify occupational processes capable of disturbing these materials and generating RCS-containing dust.

These three components were evaluated together to identify sectors and occupational tasks with potential for RCS exposure. Sector level workforce statistics were not considered evidence of actual occupational exposure. In the absence of task specific exposure measurements or robust exposure assessments, workforce statistics were used only to characterize the size of populations employed in sectors where RCS-generating activities may occur. Evidence of demonstrated occupational exposure was distinguished from potential exposure when quantitative exposure measurements or other robust exposure assessment data were available.

Because Saudi specific quantitative exposure data were limited and the included evidence varied considerably in study design, industrial setting, population, exposure assessment methods, and geographical context, the findings were synthesized narratively rather than pooled into a quantitative national exposure estimate. Where evidence differed across studies, differences in occupational tasks, industrial processes, exposure assessment methods, and geographical settings were considered when interpreting the findings. The review therefore aimed to characterize potential occupational RCS exposure and current evidence gaps, rather than estimate the prevalence or national burden of occupational RCS exposure in Saudi Arabia.

## 3 The Silica Hazard Triangle framework

In the absence of comprehensive exposure assessment data, a preliminary understanding of potential occupational exposure to RCS in Saudi Arabia can be developed by considering three interacting components: workers, silica-containing materials, and activities capable of generating RCS dust. The Silica Hazard Triangle integrates these established components into a simplified RCS specific framework for hazard identification. Its purpose is not to demonstrate actual exposure or quantify health risk, but to identify circumstances in which occupational RCS exposure could potentially occur. Accordingly, the presence of workers in a sector does not establish exposure, the presence of silica-containing material does not necessarily result in respirable exposure, and a silica-generating activity does not necessarily result in hazardous exposure, particularly where effective controls are implemented.

The convergence of the three components indicates the potential for occupational exposure but does not establish the presence or magnitude of actual exposure. Exposure may be substantially reduced or prevented by effective control measures, including process modification, wet methods, enclosure, ventilation and local exhaust ventilation, housekeeping practices, work organization, and respiratory protection. Therefore, the Silica Hazard Triangle is intended as a preliminary hazard identification framework that complements, rather than replaces, established exposure assessment and risk management approaches, including the Hierarchy of Controls. Quantitative exposure assessment remains necessary to determine actual exposure levels and characterize associated health risks.

### 3.1 Workers

The first step in identifying the potential for occupational RCS exposure in Saudi Arabia is to analyze the distribution of the workforce across industrial sectors. This section analyzes workforce distribution using the most recent publicly available labor statistics from the GASTAT, DataSaudi, and open-data portals such as the Business Atlas of the Monsha’at. These data indicate that many workers are employed in sectors involving activities associated with the generation of airborne RCS particles.

According to the Mineral Resources Statistics 2024 report, 5,651 mining sites have been identified in Saudi Arabia, of which 54.1% are associated with nonmetallic minerals and 42.9% with metallic minerals (20). Nonmetallic deposits, such as silica sand, quartz, and sandstone, may contain high concentrations of crystalline silica. Therefore, operations at these sites involving extraction, crushing, transportation, and processing can generate substantial amounts of airborne RCS dust, increasing the potential for occupational exposure among workers engaged in these tasks. Similarly, operations in metallic mines may also generate RCS in varying concentrations when silica-containing rock is extracted, crushed, or processed. The continued development of mining sites and expansion of employment in these sectors present occupational health challenges that require monitoring and targeted controls for specific high-risk tasks.

According to 2025 Business Atlas data from Monsha’at, several major employment sectors in Saudi Arabia are directly linked to activities that can generate RCS dust. In the construction sector (International Standard Industrial Classification of All Economic Activities (ISIC) Level 1), the number of employees reached approximately 3,433,993 in 2025, representing an increase of approximately 5% compared with 2024. This indicates that construction workers constitute a potentially high-risk group because of the widespread performance of activities such as concrete cutting, drilling, trenching, demolition, and tunneling.

Employment in the mining and quarrying sector (ISIC Level 1) reached 204,513 workers. Within the “other mining and quarrying” category (ISIC Level 2), 30,057 workers were engaged in activities such as stone, sand, and aggregate extraction, during which blasting and crushing can generate substantial amounts of RCS dust. Furthermore, the mining of coal and lignite sector employed 34,665 workers, while the mining of metal ores sector employed 4,342 workers. The extraction of crude petroleum and natural gas employed 82,407 workers and may also present silica exposure risks through silica sand handling, upstream drilling, and work-over operations. In addition, the transportation and storage sector employed 837,655 workers, some of whom may be indirectly exposed during the loading, unloading, transportation, and storage of silica-containing materials.

The manufacturing sector (ISIC Level 1) employed 1,494,515 workers and included numerous silica-generating processes, including cement and concrete production, metal casting, foundry operations, engineered stone fabrication, glass and ceramic manufacturing, and abrasive production. Within this category, “other manufacturing” (ISIC Level 2) accounted for 27,547 workers, some of whom may be employed in smaller facilities with limited exposure controls.

Collectively, millions of workers are employed in sectors in which RCS dust may be generated (21), highlighting the potential scale of occupational exposure and the critical need for systematic exposure assessment, monitoring, and preventive control strategies. Therefore, comprehensive regulatory frameworks and worker protection measures are urgently needed across the Saudi labor market.

### 3.2 Material

The second aspect to consider when assessing workers’ potential exposure to RCS is the chemical and mineralogical composition of the materials and substances handled in the industrial sectors described above. The presence of a significant proportion of crystalline silica in a material provides an important indication of its potential hazard.

Silicate minerals are the most abundant mineral group in the Earth’s crust, accounting for more than 90% of rock-forming minerals because of the high abundance and chemical affinity of silicon and oxygen, with quartz being a major constituent of this group (22, 23). Shadfan et al. (24), characterized the mineralogical composition of Saudi Arabian soils and identified quartz, a form of crystalline silica, as a predominant mineral component. In Saudi Arabia, petrographic analyses of sand deposits in the Tabuk area have shown that local sands are composed of approximately 98% quartz (silicon dioxide, SiO₂), indicating that crystalline silica is a dominant mineral in indigenous soil and sand materials before industrial disturbance or processing (25). This proves that crystalline silica existed in the material source inherently long before any industrial disturbance. Hazardous exposure occurs primarily when anthropogenic activities, such as mining, construction, and materials processing, disturb naturally occurring quartz and generate particles within the respirable size fraction.

The rapid expansion of silica sand production, also known as industrial sand production, and downstream silica-based industries in Saudi Arabia has important implications for public and occupational health because extraction, processing, and industrial utilization may increase potential exposure to RCS. Silica sand production in Saudi Arabia has increased by approximately 350% since 2003 (26). The mining and metals sector has been positioned as the “third pillar” of the national industrial base. This reflects the Kingdom’s substantial support of nonmetallic minerals and the strategic role of mining under Saudi Vision 2030 in supporting industrial diversification (27). National market analyses project continued growth in silica sand demand, with consumption expected to increase from approximately 0.7–0.8 million metric tons in the mid-2020s to more than 1.3 million metric tons by the mid-2030s, corresponding to a compound annual growth rate (CAGR) of approximately 6.3%. This growth is driven mainly by demand from the construction materials sector, glass manufacturing, foundry applications, water infrastructure, and high-purity silica applications associated with renewable energy technologies (28). This expansion may result in a corresponding increase in occupational exposure across mining, beneficiation, transportation, construction, and manufacturing settings.

Within this context, the *Minerals Inventory and Potential Assessment Report* (26) documents that Saudi Arabia contains billions of tons of silica sand resources, with high-purity silica sand resources containing more than 97.5% SiO₂ estimated at approximately 1 billion tons. The report also indicates that exploration and extraction activities have been strategically concentrated in several key geological formations (see Figure 2), including the Saq Sandstone Formation, shown in dark orange, in the Tayma-South and Tabuk regions (25); the Qasim Formation, shown in green, in the Qassim–Buraydah area; the Sarah Formation, also shown in green, in the Jabal al Fuhah–Wadi al Haddat region; the Biyadh Formation, shown in purple, in the Al Butayn–Al Kharj corridor; and the Wasia, or Sakaka Sandstone, Formation, shown in dark blue, in the Al Jawf–Sakaka area (26). These formations exhibit favorable grain-size distributions, high quartz purity, and low concentrations of iron oxides and alumina, making them suitable for glass, foundry, chemical, and other high-value industrial applications within the Kingdom. From public and occupational health perspectives, the geographical distribution of these deposits, together with the urban expansion across the Kingdom, means that many deposits are located near urban areas, industrial sites, and infrastructure. Silica extraction and processing may therefore affect increasing numbers of workers and nearby communities, emphasizing the importance of exposure assessment, land use planning, and environmental dust control to mitigate occupational and ambient air quality risks.

**Figure 2:**
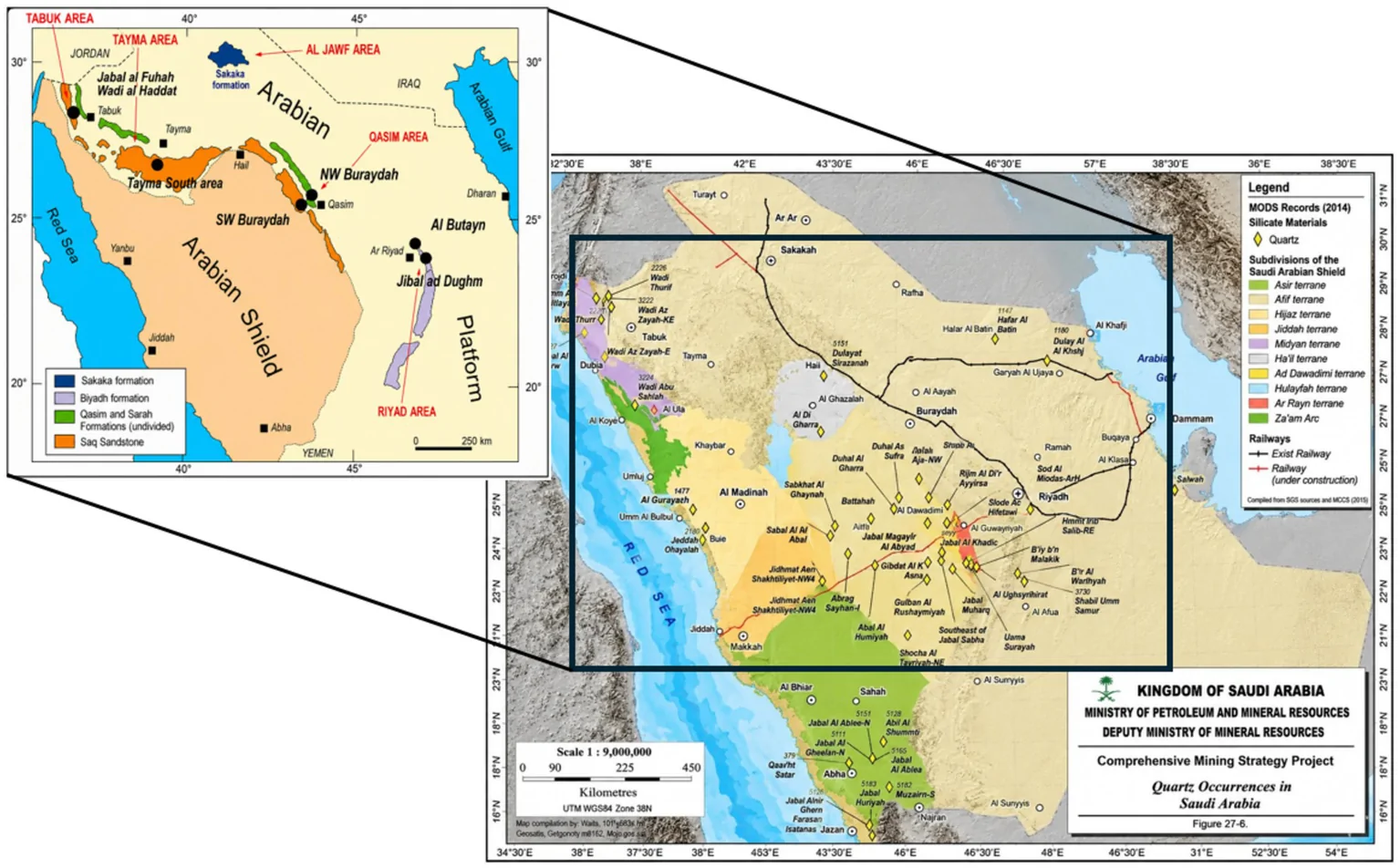
Enhanced and integrated map of silica potential and sites in the Kingdom of Saudi Arabia (from Hains et al., 2016 (26)).

The report also highlights that silica sand quality requirements differ substantially according to end use, with high-purity and specialty applications requiring additional processing steps such as washing, attrition scrubbing, classification, and beneficiation. These processes mechanically disturb silica-bearing materials and are therefore important determinants of airborne RCS generation. The report also discusses industrial applications relevant to the Saudi context, together with the silica purity requirements for each industry (see Table 1) (29).

**Table 1:** Industrial application specifications for silica sand.

Industrial applications	Indicative requirements					
	Chemical composition (percent)				Grain size	Remarks
	Min SiO_2_	Max Al_2_O_3_	Max Fe_2_O_3_	Miscellaneous		
Glass						
Container colored Container colorless Flat Colorless domestic Optical Glass wool Continuous filament	98.5 98.5 98.5 99.5 99.5 95.0 99.0	↑ 0.1 ~0.5 ↓ 1.7	0.18 0.03 0.03 0.013 0.008 0.3 0.1		0.1–0.5 mm 0.1–0.5 mm	Less than: 6 ppm Cr 2 ppm Co 0.01–0.5% TiO_2_
Foundry sand	85 (iron) 95 (steel)				20–200 mesh	Sub-angular to rounded grains
Ceramics	97.5	0.55	0.2		–200 mesh	
Hydraulic fracturing	98			<0.3% solubility in HCl	20–40 mesh 0.4–0.8 mm	Rounded particles (Krumbein roundness factor ≥0.6)
Filtration	98			<0.2% solubility in HCl 20%	0.8–2 mm	Rounded and uniform grains
Fillers	97				micron size	• Brightness 53–89 • Oil absorption 13–50
Silica flour					−60 μm	
Sodium silicate	99.4	0.25	0.03	max CaO/ MgO 0.05	20–100 mesh	
Abrasives					4–8 mesh (iron and steel) 20–35 mesh (light work)	Angular to rounded particles
Silicon carbide	99.5	0.06–0.25	0.1		micron size	No phosphorus allowed
Silicon	99	0.2–0.3	0.1–0.2	0.1 CaO, 0.1 MgO, 0.02 TiO_2_		

Al₂O₃, aluminum oxide; CaO, calcium oxide (quicklime); Co, cobalt; Cr, chromium; Fe₂O₃, ferric oxide; HCl, hydrochloric acid; MgO, magnesium oxide; SiO₂, silicon dioxide (quartz); TiO₂, titanium dioxide.

Saudi Arabia is pursuing investment opportunities to produce up to 144 kilotons per year of high-purity silica sand for domestic markets in support of Saudi Vision 2030, particularly for glass, silicon metal, and advanced manufacturing. As a result, the intensity of beneficiation and materials handling operations is expected to increase (30). Without appropriate engineering controls, exposure monitoring, worker training, and medical surveillance, this expansion may increase occupational health risks despite its economic benefits. Therefore, silica processing efficiency and product specification control should be integrated with occupational health risk management.

From a broader public health perspective, continued expansion of silica-related industries highlights the need for proactive land use regulation, environmental management, and workforce protection frameworks. These considerations are further reinforced by Saudi Arabia’s national target of 58.7 GW of renewable energy capacity by 2030, which is expected to increase demand for silicon, a key input in PV cells, as well as aluminum–silicon alloys used in construction, transportation, and energy systems. Saudi Arabia is also planning to establish localized PV manufacturing facilities to reduce reliance on imports (30).

As silica production and downstream silicon industries expand to meet these strategic objectives, failure to protect high-quality deposits from urban encroachment and control dust emissions during mining, construction, transportation, and processing may increase population-level exposure risks. Collectively, these findings support a preventive occupational and environmental health approach. Integrating mineral resource planning with occupational health regulations may help reduce the burden of silica-related disease while supporting economic diversification and energy transition goals.

Environmental and climatic conditions may influence the generation, transport, and resuspension of airborne dust in Saudi Arabia. The Kingdom’s arid climate and limited precipitation may favor the persistence and resuspension of dust; however, the presence of airborne dust alone does not establish occupational exposure to RCS. Total airborne dust includes particles across a broad range of sizes and compositions, whereas occupational RCS exposure specifically concerns the respirable fraction containing crystalline silica that can penetrate into the gas-exchange region of the lungs. Therefore, potential health risk depends on several factors, including particle size, the crystalline silica fraction of the respirable dust, airborne concentration, and the duration and frequency of exposure. Quantitative measurements of respirable dust and its crystalline silica content are therefore necessary before occupational RCS exposure or associated health risk can be inferred among outdoor workers in Saudi Arabia.

Environmental and climatic influences on dust distribution must also be considered. Statistical reports published by the Ministry of Environment, Water and Agriculture in 2023 indicated that the average annual precipitation in Saudi Arabia is approximately 106 mm, which is considerably lower than that of countries such as the United States and the United Kingdom, where average precipitation levels are approximately sevenfold and twelvefold higher, respectively (31, 32). Precipitation in most regions of Saudi Arabia is scarce and infrequent and occurs mainly between October and April. The remaining months experience almost no rainfall, except in the southwestern mountainous regions. The historical average annual precipitation across Saudi Arabia between 1979 and 2009 was approximately 93.5 mm (33).

Such arid climatic conditions may contribute to the generation and prolonged suspension of silica-containing particles in ambient air because limited precipitation reduces wet deposition and may facilitate the resuspension and aerosolization of dust compared with regions characterized by higher rainfall. Consequently, some potentially overlooked occupational groups, such as graveyard workers and street-cleaning workers, may be exposed to silica-containing dust. However, research evaluating RCS exposure and associated health risks among these occupational groups remains limited.

### 3.3 Activities

All the industries discussed below are major contributors to the Saudi economy. At the same time, the literature has documented associations between specific industrial activities (tasks) and occupational silica exposure at both regional and international levels. Occupational exposure to freshly fractured or newly generated crystalline silica during processing and fabrication may be more hazardous than exposure to aged or environmental silica because freshly generated particles can have greater surface reactivity and may trigger more intense inflammatory responses in lung tissue. However, the presence of silica-containing dust, whether from environmental or industrial sources, does not by itself establish RCS exposure. The potential health risk depends on the respirable fraction of the dust, its crystalline silica content, airborne concentration, and the duration and frequency of exposure. Therefore, environmental dust should not be considered inherently harmless, while industrial activities that mechanically disturb silica-containing materials may generate higher concentrations of RCS and require quantitative exposure assessment (34, 35).

#### 3.3.1 Oil and gas (hydraulic fracturing)

International evidence demonstrates workers in hydraulic fracturing (fracking) operations are exposed to elevated levels of RCS, which significantly increases the risk of silicosis and other respiratory diseases (36). During hydraulic fracturing, large volumes of quartz-containing sand are used as proppants. Field investigations conducted across multiple U.S. states found that airborne RCS concentrations frequently exceeded OELs, in some cases by up to tenfold, particularly during sand handling, transfer, and other site operations.

Exposure during hydraulic fracturing occurs primarily during tasks involving sand handling, transfer, and processing. Workers operating sand movers, blender systems, and conveyor belts (T-belts), experience high exposure because of the continuous mechanical agitation and release of silica-containing dust. Additional exposure may occur during equipment operation and other site activities, particularly among workers located near or downwind of dust-generating processes (37). In the Saudi context, oil and gas service companies conducting hydraulic fracturing for unconventional gas production and the development of tight gas reserves represents a potential exposure pathway requiring investigation, particularly during sand handling, transfer, and processing activities.

#### 3.3.2 Cement industry

Occupational exposure to RCS in cement manufacturing is associated with an increased prevalence of chronic respiratory symptoms, including cough, phlegm, wheezing, and shortness of breath. Exposed workers experience reduced lung function and a higher risk of respiratory impairment compared with non-exposed groups (38). Consistent with these findings, regional studies conducted in Iran have demonstrated that cement workers are exposed to crystalline silica concentrations exceeding OELs, with associated impairments in pulmonary function (39).

Similar patterns have been observed in Saudi Arabia. Although Saudi studies have primarily focused on general cement dust exposure rather than specifically quantifying RCS, cement workers in the Qassim region were reported to have a high prevalence of respiratory complications and inflammatory changes following chronic occupational dust exposure (40). These findings suggest that industrial dust exposure, potentially including crystalline silica-containing particles, may contribute to adverse respiratory health outcomes among cement industry workers in Saudi Arabia.

#### 3.3.3 Ceramic industry and engineered (artificial) stone

Ceramic manufacturing and processing activities, such as cutting and grinding, are well documented sources of occupational exposure to RCS (41). Specific tasks, particularly granulation and glazing, present some of the highest exposure risks, highlighting the need for effective exposure controls and occupational health measures (42). Recent regional evidence from Iran further supports these findings, showing that workers in silica-related industries, including ceramics, are exposed to RCS concentrations exceeding international OELs and face substantially elevated health risks (13).

The type of material used in a process is a key factor influencing the potential for silica exposure. While natural stones such as limestone and marble, which are calcium carbonate-based, contain little or no crystalline silica, granite can contain up to 50% crystalline silica. Furthermore, quartzite and certain engineered stones contain high levels of crystalline silica, sometimes exceeding 90%. In engineered stone, crystalline silica can account for up to 95% of the material, with resins, pigments, and adhesives making up the remaining percentage.

Artificial stone has increasingly become a substitute for natural stone because of economic considerations. This has contributed to its growing popularity in construction applications, including commercial and residential countertops (43). The increasing use of artificial and engineered stone products in residential and commercial construction, particularly for countertops and interior surfaces, has raised significant occupational health concerns.

Recent studies have highlighted the increased prevalence of silicosis among workers involved in the manufacturing, finishing, and installation of engineered stone countertop products. Workers in fabrication workshops and at construction or installation sites commonly perform tasks such as sawing, grinding, polishing, and drilling engineered stone slabs (man-made, manufactured, and artificial), all of which can generate substantial concentrations of airborne RCS dust (44).

Moreover, recent findings from 2026 demonstrate that artificial stone fabrication is one of the most hazardous occupational settings for RCS exposure and contain extremely high levels of crystalline silica, with widespread reports of accelerated silicosis, severe lung disease, and increasing mortality among exposed workers (45, 46). In response, OSHA has emphasized its National Emphasis Program (NEP) on RCS to reduce worker exposure, particularly in industries involving engineered stone fabrication and countertop manufacturing, where some measurements have reached nearly 20 times the OSHA exposure limit. OSHA inspections have also revealed deficiencies in respiratory protection, hazard communication, and medical surveillance programs among employers (47).

Further investigation is warranted in the Saudi Arabian context, particularly among workers employed in ceramic manufacturing and engineered stone fabrication, because these industries involve tasks with well established potential for RCS exposure internationally.

#### 3.3.4 Mining and quarry operations

Mining operations in underground and quarry settings inherently generate dust, including RCS, because crystalline silica is a major component of the Earth’s crust. Therefore, mining represents a major source of occupational exposure (3). Metallic and nonmetallic mining are considered high-risk occupational settings for RCS exposure, with documented exposure levels frequently exceeding occupational limits (48).

Multiple peer-reviewed studies, including regional studies, have indicated that workers in the mining sector are exposed to RCS during operational activities such as drilling, crushing, and material handling. These activities increase the risk of silicosis, lung cancer, and other respiratory diseases (12, 49). The NIOSH has developed comprehensive guidance, analytical methods, and exposure-monitoring tools to protect mine workers (50). Stone cutters and quarry workers are recognized as high-risk occupational groups, particularly in open quarries and stone processing environments, where prolonged work shifts and repetitive handling of silica-containing materials contribute to increased cumulative inhalation exposure to RCS.

Recent evidence further demonstrates that RCS exposure within the mining sector is not uniform across operations. Siahidouzazar et al. (51), analyzing nearly 65,000 samples collected from U.S. metal and nonmetal mines between 2000 and 2023, found that RCS concentrations frequently exceeded permissible exposure limits (PELs) and identified significant variation according to mine type, mine size, geographic location, and mine category. Higher concentrations were particularly evident in large surface mines, while metal and nonmetal mines generally presented greater silica hazards than sand and gravel and stone operations. These findings demonstrate the importance of considering mine and operation specific characteristics when assessing occupational RCS exposure.

These findings have important implications for Saudi Arabia, where investment in the mining sector has recently been prioritized as part of Saudi Vision 2030. As the sector continues to expand, future national exposure assessments should therefore account for mine type, operation size, geographic location, mineral characteristics, and specific dust-generating tasks when identifying workers and tasks with the potential for RCS exposure and prioritizing them for exposure monitoring and risk assessment.

#### 3.3.5 Construction

Construction activities are a well established source of occupational exposure to RCS, particularly during tasks such as concrete cutting, drilling, grinding, and demolition. Multiple peer-reviewed studies have demonstrated that a large proportion of construction workers are exposed to RCS at levels exceeding OELs, placing them at increased risk of silicosis, lung cancer, and other respiratory diseases (52, 53).

For example, exposure assessments in road construction have reported RCS concentrations reaching up to 30 times OELs, highlighting the severity of exposure during specific job tasks (54). In addition, epidemiological and exposure database studies confirm that silica exposure is widespread across construction trades and represents a persistent occupational health hazard (53).

The OSHA has identified construction as one of the highest risk industries for silica exposure, emphasizing that materials such as concrete, brick, and mortar contain significant amounts of crystalline silica and generate hazardous dust during mechanical processing (55). In Saudi Arabia, the construction sector employs one of the largest workforces and continues to expand under national development plans, thereby representing a substantial population potentially exposed to RCS during specific dust-generating tasks. However, the proportion of construction workers with actual RCS exposure has not been established through national exposure monitoring.

#### 3.3.6 Glass manufacturing

Glass manufacturing involves the extensive use of quartz sand as a primary raw material, which can generate RCS dust. Processes such as raw material handling, preparation, and melting can release quartz-containing dust into the workplace atmosphere, leading to occupational exposure among workers.

Studies conducted in glass production facilities have demonstrated that workers in batch preparation, material charging, and unloading areas are exposed to silica dust concentrations exceeding OELs, especially in areas involving the loading, mixing, and transportation of silica-containing materials (56). Furthermore, early stage processes such as crushing and milling silica raw materials used in glass production generate high levels of airborne quartz particles, contributing to an increased risk of silicosis and other respiratory diseases among exposed workers (57).

The development of silicosis is strongly influenced by the intensity and duration of exposure. Evidence from an Iranian study indicates that silicosis can develop within 1–15 years of occupational exposure and within as little as 1–3 years in high-exposure environments, such as silica powder industries (57). This evidence highlights that short-term, high-intensity occupational exposure in glass manufacturing can result in the development of advanced silicosis.

Conversely, a case report from Korea described a glass plant worker with more than 20 years of employment whose job tasks involved the manual handling and mixing of silica-containing raw materials and who developed complicated silicosis. This case underscores that long-term occupational exposure in glass manufacturing, even at relatively moderate concentrations, can also lead to advanced disease (58).

#### 3.3.7 Photovoltaic manufacturing

Photovoltaic manufacturing involves multiple processes that could cause occupational exposure to silica-containing materials, particularly during the production and handling of silicon wafers derived from quartz. Crystalline silicon, which is used in most PV cells, is produced from high-purity silica through high temperature reduction and additional processing steps, including crushing, cutting, grinding, and wafer slicing. These activities can generate RCS dust.

Workers involved in raw material handling, silicon refining, wafer fabrication, and equipment maintenance are at potential risk of exposure to silica dust, especially during upstream processes in which quartz is processed into purified silicon.

In addition to particulate exposure, PV manufacturing environments involve exposure to other hazards, including toxic gases and chemical by-products, as highlighted in occupational health reviews of the industry (59). Therefore, PV manufacturing presents a complex occupational exposure profile involving both silica-containing particulate matter and other chemical hazards.

Photovoltaic panels pose no silica-related inhalation risk during normal use, yet silica-containing dust generated during manufacturing is recognized as a significant respiratory hazard. Therefore, silica-related risks are concentrated primarily in manufacturing environments and require appropriate industrial hygiene controls (60).

In Saudi Arabia, PV manufacturing is expanding in response to renewable energy initiatives. These exposure pathways highlight the need for effective exposure monitoring and control strategies.

To provide a concise overview of the occupational settings discussed throughout this review, Table 2 summarizes the principal industries associated with potential RCS exposure in Saudi Arabia. The table outlines the major exposure-generating tasks, the primary silica-containing materials handled, and the estimated workforce employed in each sector where data are available.

**Table 2:** Potential at risk industries and occupational exposure pathways for respirable crystalline silica (RCS) in Saudi Arabia.

Industry	Major exposure pathways/tasks	Main silica-containing materials	Total sector workforce* (not equivalent to RCS-exposed workforce)
Construction	Concrete cutting, drilling, grinding, demolition, trenching, tunnelling	Concrete, cement, brick, mortar, stone	3,433,993
Manufacturing	Cement production, glass manufacturing, ceramics, foundries, engineered stone fabrication, abrasive manufacturing	Cement, quartz, silica sand, engineered stone, glass raw materials	1,494,515
Mining and quarrying	Drilling, blasting, crushing, screening, excavation, material handling	Quartz, silica sand, sandstone, rock	204,513
Oil and gas	Hydraulic fracturing, silica sand handling, transfer, blending and storage	Silica sand (proppant)	82,407
Transportation and storage	Loading, unloading, transport and storage of silica-containing materials	Bulk silica sand, aggregates, industrial minerals	837,655
Glass manufacturing	Batch preparation, charging, crushing, mixing and raw material handling	High-purity silica sand	Included within manufacturing
Ceramics and engineered stone	Cutting, grinding, polishing, finishing and fabrication	Ceramic materials, engineered stone, quartz	Included within manufacturing
Photovoltaic manufacturing	Silicon refining, wafer cutting, grinding and material handling	High-purity silica (SiO₂)	Included within manufacturing

^**^Total sector workforce populations are based on 2025 Business Atlas (Monsha’at) employment statistics where available. These figures represent total employment within each sector and should not be interpreted as estimates of the number of workers exposed to RCS. Glass manufacturing, ceramics, engineered stone fabrication, and photovoltaic manufacturing are included within the broader manufacturing sector and therefore do not have separate workforce populations.

## 4 Established regulatory exposure limits for RCS

RCS is a well established and recognized occupational carcinogen (61) and a leading cause of silicosis, COPD, and lung cancer. This evidence has led many industrialized countries to adopt increasingly stringent OELs. For instance, in the United States, after 45 years OSHA implemented the revised silica standard on June 23, 2016, establishing a PEL of 50 μg/m^3^ compared to the previous 100 μg/m^3^ (8-h TWA) and requiring continuous exposure monitoring, engineering controls, and medical surveillance across general industry, maritime, and construction sectors (62).

In 2023, OSHA also initiated a National Emphasis Program targeting high-risk occupational exposure to RCS in response to the increasing burden of silicosis and associated diseases. (47) Complementing these measures, the MSHA issued an update to its silica regulations, effective April 18, 2024, lowering the RCS PEL for miners to 50 μg/m^3^ (from the previous limit of 100 μg/m^3^) and strengthening enforcement mechanisms and health protections specific to mining and quarrying operations (63).

Table 3 summarizes the historical regulatory exposure limits for RCS and presents the current science-based OELs established by major regulatory and advisory organizations in the United States. These regulatory actions reflect the growing body of scientific evidence in public and occupational health, beginning in the early 1960s, demonstrating adverse health effects at lower exposure levels. They also exemplify international progress toward stronger respiratory protection for workers (4).

**Table 3:** Regulatory exposure limits for respirable crystalline silica (RCS).

Source / Authority	Limit / Value	Time averaging	Purpose / Basis /Comments
OSHA (pre- 2016)	100 μg/m^3^ (0.1 mg/m^3^) for quartz	8-h TWA	The older PEL for quartz under general industry rules (4).
OSHA (2016- present)	50 μg/m^3^ (0.05 mg/m^3^)	8-h TWA	New (2016) standard for RCS. In 2023, OSHA established an emphasis program on silica in engineered stone (47, 62).
NIOSH (REL)	50 μg/m^3^ (0.05 mg/m^3^)	8-to-10-hourTWA	Health-based recommendation intended to prevent silicosis and lung disease (73).
ACGIH (TLV)	25 μg/m^3^ (0.025 mg/m^3^)	8-h TWA	ACGIH lowered its TLV (for quartz) to 0.025 mg/m^3^ in recognition of risk at lower exposures (74).
MSHA (pre-2024)	100 μg/m^3^ (0.1 mg/m^3^)	8-h TWA	Scientific epidemiological studies showed that RCS-related diseases are still observed at this limit of exposure (48).
MSHA (2024- present)	50 μg/m^3^ (0.05 mg/m^3^)	8-h TWA	2024 Final Rule aligning mining silica OELs with federal standards to reduce miners’ exposure and prevent silicosis (63).

ACGIH, American Conference of Governmental Industrial Hygienists; OEL, occupational exposure limit; PEL, permissible exposure limit; REL, recommended exposure limit; RCS, respirable crystalline silica; TLV, threshold limit value; TWA, time-weighted average. Occupational exposure limit terminology varies among regulatory and scientific organizations. PELs are legally enforceable regulatory limits within the applicable jurisdiction, whereas RELs and TLVs are health-based recommendations or guidelines developed by NIOSH and ACGIH, respectively. These limits may also differ according to the exposure duration over which concentrations are assessed, such as an 8-h TWA.

As Saudi Vision 2030 drives the rapid expansion of silica-intensive industries, including construction, mining and quarrying, glass and ceramic manufacturing, engineered stone fabrication, and unconventional oil and gas development, a growing number of workers may perform tasks with potential for RCS exposure. In this context, recent international regulatory developments, including OSHA’s 2016 revised silica standard, its 2023 National Emphasis Program for RCS, and MSHA’s 2024 regulatory update, highlight the need for clearly defined and enforced national exposure limits based on current scientific evidence and international best practices.

Establishing health-based PELs and recommended exposure limits (RELs) for RCS is a critical regulatory priority to ensure that economic growth and industrial diversification do not result in a preventable future burden of occupational respiratory disease in the Kingdom.

## 5 Legal context and governance of regulatory exposure in Saudi Arabia

As of May 23, 2026, information regarding specific Saudi regulations on silica exposure remains limited. However, the policy of the Saudi NCOSH emphasizes the adoption of international best practices. Consequently, major enterprises in industries such as oil and gas, chemical manufacturing, construction, and mining commonly adopt exposure limits and occupational hygiene guidance from internationally recognized organizations, including the United States’ OSHA, NIOSH, the American Conference of Government Industrial Hygienists (ACGIH) and the United Kingdom’s Health and Safety Executive (HSE).

Although Saudi Arabia has not yet established nationally published OELs or silica specific regulations for RCS, the existing legal and institutional framework provides a strong foundation for protecting workers from occupational hazards. The purpose of the following discussion is therefore not to evaluate silica specific legislation, which does not currently exist, but to examine the broader legislative and institutional framework that would support the future development and implementation of silica-specific regulations, OELs, occupational hygiene programs, and disease surveillance. In this context, the current framework already establishes employer responsibilities and general occupational health principles that could facilitate the incorporation of internationally recognized best practices for the prevention and control of RCS exposure.

The Saudi Labor Law which was issued by Royal Decree No. M/51 on 3/8/1426 AH and amended by Royal Decree No. M/44 on 8/2/1446 AH. Part 8, Chapter 1, titled “Protection from Occupational Hazards,” outlines employers’ legal duties to ensure a safe working environment (64) akin to occupational health and safety legislation and regulations in many countries.

The Saudi Labor Law mandates that every employer provide both preventive and therapeutic (curative) healthcare to employees. It also serves as a legal link between the Labor Law (65) and the Cooperative Health Insurance Law (66), with GOSI responsible for treatment and rehabilitation costs, wage replacement, and compensation for permanent or total disability related to silica-associated health conditions.

NCOSH was established in 2022 in coordination with the MHRSD and supports the development and updating of national occupational safety and health standards, regulations, and technical guidelines to operationalize the Saudi Labor Law and ILO Conventions 155 and 187. However, this broader regulatory role should be distinguished from the existence of a silica specific exposure standard; as of the review date, a nationally published, legally enforceable RCS-specific OEL was not identified in the official Saudi sources reviewed. The national policy applies to all workplaces and sectors, ensuring equal protection for all workers. It prioritizes workers’ right to perform their duties without harm. The policy adheres to principles of prevention that directly relate to safeguarding, preserving, and enhancing the physical, mental, and social well-being of workers. It also involves the development of legislation, systems, bylaws, regulations, and other regulatory tools specifically designed to address occupational safety (67).

Recently published fitness-for-duty guidelines issued by NCOSH include questions regarding major occupational hazards, including silica exposure (68). They also include questionnaires and medical assessments, including imaging, for periodic physical examinations of workers in high-risk occupations such as construction and mining. In addition, specific investigations for silica-exposed workers are integrated into the risk assessment process. Finally, all exposed workers undergo an end-of-service assessment (exit medical examination) to determine whether any long-term disability is present.

Silica exposure is explicitly addressed in the Saudi Schedule of Occupational Diseases issued by GOSI, which draws heavily from the ILO 2010 List (69). Silica-related diseases are classified under “Occupational Respiratory Diseases,” Codes 2.1.1 and 2.1.2, and under the “Occupational Cancer” section, Code 3.1.

According to the most recent schedule update issued by GOSI in 2022, silica and other agents that cause pneumoconiosis are recognized as occupational hazards. The document classifies diseases caused by mineral dusts under occupational respiratory diseases, with SiO₂ listed as a causative agent of serious lung conditions such as pneumoconiosis and silicosis. The schedule recognizes that long-term exposure to RCS dust can lead to irreversible fibrotic lung disease. In addition, it highlights that exposure assessment and occupational history are essential for diagnosis and compensation decisions under the Saudi occupational hazards framework (70). Overall, the Saudi occupational disease schedule aligns with the international evidence by recognizing silica as a major occupational hazard and clearly defining its health effects for compensation and prevention purposes.

Article 131 of the Saudi Labor Law emphasizes that: “The Minister shall issue the regulations and decisions that include the necessary arrangements at firm level for protection against major hazards…”. Although specific national exposure limits for RCS have not yet been formally established in Saudi Arabia, the existing labor, health, and social insurance regulations clearly acknowledge the occupational health risks associated with silica exposure. Together, these frameworks demonstrate a strong commitment to worker protection, disease prevention, and regulatory alignment with international occupational health standards, such as those established by OSHA and the ILO.

Recently, licenses for silica processing in Saudi Arabia have been temporarily suspended through regulatory directives as part of economic restructuring. However, this is considered an emerging valuable opportunity to re-evaluate and strengthen the national occupational health and safety standards and regulations (71). The optimal approach in the Saudi Arabian context is to capitalize on the economic value of silica resources while implementing better controlled workplace environments. Applying industrial hygiene standards and enhanced monitoring techniques can assist Saudi Arabia in strengthening its current and future occupational safety practices and standards.

Several important aspects of occupational health remain poorly characterized in Saudi Arabia, including national occupational exposure surveillance systems, disease surveillance programs, compensation statistics for silica-related diseases, and the economic burden associated with occupational RCS exposure. Similarly, limited published evidence is available regarding potentially vulnerable worker groups, such as migrant workers and employees of small enterprises, who may experience different exposure profiles and levels of access to occupational health services. Addressing these knowledge gaps through national surveillance programs, epidemiological studies, and economic evaluations will be essential for quantifying the prevalence and magnitude of occupational RCS exposure and the associated health burden in Saudi Arabia.

Recent international monitoring evidence further demonstrates the importance of developing a risk-based national surveillance system rather than relying solely on general occupational exposure requirements. Chu et al. (72), in a 5-year study of industrial and mining enterprises in Sichuan Province, China, reported an overall silica-dust compliance rate of 82.2%, with significant improvement between 2021 and 2025. However, important differences persisted according to industry, geographic region, enterprise scale, and free silica content. These findings are particularly relevant to Saudi Arabia, where comprehensive national RCS exposure-monitoring data remain limited. A future Saudi surveillance framework should therefore stratify exposure assessment and preventive interventions according to sector, specific occupational tasks, silica content of materials, geographic location, and enterprise characteristics. Such a risk-based approach could help identify high-exposure workplaces, prioritize monitoring and enforcement resources, and guide targeted engineering controls, worker protection, and medical surveillance.

Table 4 summarizes the main Saudi legal provisions and institutional mechanisms relevant to occupational RCS exposure, highlighting employer responsibilities, medical surveillance, disease recognition, and the remaining regulatory gaps.

**Table 4:** Summary of the legal and institutional framework relevant to occupational respirable crystalline silica (RCS) in Saudi Arabia.

Regulatory authority / Source	Legal or institutional instrument	Relevant provision or function	Relevance to occupational RCS exposure	Current limitation or implication
Saudi Labor Law (General duty)	Part 8, Chapter 1	Establishes employers’ general legal duties to protect workers from occupational hazards and provide a safe working environment.	Provides the primary legal basis for preventing and controlling workplace exposure to silica dust.	Does not currently establish a specific national OEL for RCS.
Saudi Labor Law (Employer duty)	Article 122	Requires employers to take necessary precautions to protect workers from hazards, occupational diseases, and unsafe machinery.	Supports the implementation of engineering controls, safe work practices, and other preventive measures for silica-generating activities.	Establishes a general duty of protection but does not prescribe silica-specific control requirements.
Saudi Labor Law (Hazard communication)	Article 123	Requires employers to inform workers of job hazards and provide appropriate personal protective equipment.	Supports hazard communication, worker education, training, and respiratory protection for workers exposed to RCS.	Detailed silica-specific requirements for training, respiratory protection programs, and exposure communication are not defined.
Saudi Labor Law (Risk classification)	Articles 129 and 130	Requires the Ministry to establish criteria for identifying high-risk establishments and employers to coordinate with the Ministry regarding their classification.	Provides a legal mechanism for classifying silica-intensive workplaces, such as mining, construction, and engineered stone facilities, as high-risk establishments.	Silica-specific classification criteria and implementation procedures require development.
Saudi Labor Law (Ministry duty)	Article 131	Authorizes the Minister to issue regulations and decisions concerning protection from major hazards at the establishment level.	Provides authority for the future adoption of enforceable RCS OEL, technical standards, and control requirements.	National RCS-specific regulations and OELs have not yet been formally established.
Saudi Labor Law (Medical surveillance)	Article 143	Requires annual medical examinations for workers exposed to occupational diseases listed under the Social Insurance Law and requires recordkeeping.	Supports periodic health surveillance of workers exposed to silica and the early detection of silicosis and other silica-related diseases.	Annual medical examinations alone are insufficient; effective silicosis surveillance also requires exposure assessment, standardized medical monitoring, longitudinal follow-up, recordkeeping, referral, and disease reporting
Saudi Labor Law (Cooperative Health Insurance Law)	Article 144	Requires employers to provide preventive and therapeutic healthcare in accordance with ministerial standards and the Cooperative Health Insurance Law framework.	Supports medical care, treatment, and rehabilitation for workers affected by silica-related occupational disease.	Coordination among employers, healthcare providers, insurers, and GOSI requires clear implementation procedures.
NCOSH	Fitness-for-Duty Guidelines	Include occupational hazard questionnaires, medical assessments, imaging, periodic examinations, and end-of-service assessments (exit medical examinations) for high-risk workers.	Provide a basis for health assessment and surveillance of workers exposed to silica in sectors such as construction and mining.	Effectiveness depends on consistent implementation, exposure-based worker classification, and standardized follow-up procedures.
GOSI (Disease recognition and compensation)	Saudi Schedule of Occupational Diseases	Recognizes silica-related respiratory diseases and occupational cancers under relevant disease classifications.	Establishes RCS exposure as a recognized occupational hazard and supports diagnosis, compensation, prevention, and occupational disease reporting.	National data on compensation claims, disease incidence, exposure history, and outcomes remain limited, and not publicly available.

## 6 Conclusion

This review provides a structured investigation of the potential risk of occupational exposure to RCS in Saudi Arabia using the Silica Hazard Triangle framework. The analysis considers three determinants: the distribution of workers across relevant industries, the mineralogical composition of silica-containing materials, and the industrial activities that generate RCS dust.

The available evidence confirms that silica related disease occurs in Saudi Arabia; however, neither the prevalence of occupational RCS exposure nor the national exposure attributable health burden can currently be quantified because comprehensive exposure monitoring, epidemiological studies, occupational disease surveillance, and compensation data are limited. The findings of this review therefore identify sectors, materials, and tasks with potential for RCS exposure rather than providing an estimate of the number of exposed or at risk workers. Comprehensive field assessments using standardized personal sampling, validated analytical methods, and systematic exposure assessment strategies are therefore needed to distinguish potentially exposed workers from those with demonstrated exposure and to characterize workers at risk. Such assessments should include representative personal respirable dust sampling, determination of crystalline silica content, and task-specific measurements where appropriate. Exposure data should also be systematically documented and, where feasible, linked with occupational health surveillance records to support future epidemiological assessment and regulatory decision-making. This will require appropriate sampling and monitoring equipment, access to accredited analytical laboratories, trained occupational hygienists, and coordinated occupational health surveillance.

International regulatory developments demonstrate a continuing shift toward more stringent RCS exposure limits, stronger engineering control requirements, medical surveillance, and targeted enforcement. Saudi Arabia already has a broad legal and institutional foundation through the Saudi Labor Law, the NCOSH, and the GOSI. These frameworks establish employer responsibilities, worker protection principles, medical examination requirements, and recognition of silica-related occupational diseases. However, nationally published RCS OELs, silica-specific technical standards, standardized compliance procedures, and comprehensive surveillance systems remain important regulatory gaps.

This review has several limitations. Saudi specific quantitative RCS exposure measurements and epidemiological evidence remain limited, requiring the use of international studies to provide context for established exposure pathways and health effects. Consequently, sector level workforce data and the identification of silica-containing materials and dust-generating activities should be interpreted as indicators of potential occupational exposure rather than evidence of actual exposure. These limitations prevent estimation of the prevalence of occupational RCS exposure or the national exposure attributable health burden in Saudi Arabia.

Saudi Arabia therefore has an important opportunity to establish a proactive national framework for the prevention and control of occupational RCS exposure while silica-intensive industries continue to expand under Saudi Vision 2030. Such a framework should combine health-based OELs, routine exposure monitoring, engineering and administrative controls, respiratory protection programs, worker education, medical surveillance, disease reporting, and enforcement mechanisms.

Finally, the prevention of silica-related disease cannot be achieved through exposure assessment alone. Effective risk reduction requires a broader risk management approach involving engineers, process managers, occupational hygienists, occupational health professionals, researchers, regulators, employers, workers, and relevant stakeholders. This step is essential for translating assessment findings into effective prevention and control strategies, while exposure assessment results provide critical guidance for identifying priorities for intervention and implementing appropriate control measures. Coordinating scientific assessment, regulatory action, workplace prevention, and health surveillance will be essential for protecting workers while supporting sustainable industrial and economic development in Saudi Arabia.
